# Glycyrrhiza Polysaccharide Inhibits Pseudorabies Virus Infection by Interfering with Virus Attachment and Internalization

**DOI:** 10.3390/v14081772

**Published:** 2022-08-14

**Authors:** Changchao Huan, Yao Xu, Wei Zhang, Bo Ni, Song Gao

**Affiliations:** 1 Institutes of Agricultural Science and Technology Development, College of Veterinary Medicine, Yangzhou University, Yangzhou 225009, China; 2 Jiangsu Co-Innovation Center for Prevention and Control of Important Animal Infectious Diseases and Zoonoses, Yangzhou 225009, China; 3 Key Laboratory of Avian Bioproduct Development, Ministry of Agriculture and Rural Affairs, Yangzhou 225009, China; 4 Institutes of Agricultural Science and Technology Development, Yangzhou University, Yangzhou 225009, China; 5 China Animal Health and Epidemiology Center, Qingdao 266011, China

**Keywords:** Glycyrrhiza polysaccharide, pseudorabies virus, antiviral activity, attachment, internalization

## Abstract

Pseudorabies virus (PRV) is one of the most important pathogens causing serious diseases and leads to huge economic losses in the global swine industry. With the continuous emergence of PRV variants and the increasing number of cases of human infection, there is an urgent need to develop antiviral drugs. In this study, we discover that Glycyrrhiza polysaccharide (GCP) has anti-PRV infection activity in vitro, and 600 μg/mL GCP can completely block viral infection. The addition of GCP simultaneously with or after PRV infection had a significant inhibitory effect on PRV. Addition of GCP at different times of the virus life cycle mainly led to the inhibition of the attachment and internalization of PRV but does not affect viral replication and release. Our findings suggest that GCP has potential as a drug against PRV infection.

## 1. Introduction

Pseudorabies (PR) is an acute infectious disease caused by the pseudorabies virus (PRV), which was first discovered in Hungary in 1902, and still causes serious disease and economic loss in the world [1,2]. In China, PRV was first isolated from cats in 1948, and has since been reported in domestic economic animals, such as pigs, cattle, and foxes [3]. Vaccination is the most common method to prevent PR [4,5]. The widespread introduction of the Bartha K61 vaccine from Hungary to China has brought the PR epidemic in China under control [6]. However, since 2011, highly pathogenic PRV variants have emerged in China, and the classic Bartha K61 vaccine does not provide complete protection [7,8]. Therefore, it is important to develop new vaccines and antiviral drugs to control the transmission of PRV variants.

The first step of the virus life cycle is viral entry. PRV entry into cells is mediated by viral glycoproteins, which can be divided into two stages: attachment and internalization. First, PRV virions attach to cells through glycoprotein C (gC) interaction with heparan sulfate proteoglycan in the extracellular matrix. Then, PRV glycoprotein D (gD) binds to specific cellular receptors [9]. Finally, PRV glycoprotein B (gB), glycoprotein H (gH), and glycoprotein L (gL) mediate an energy- and temperature-dependent fusion of the viral envelope and cell membrane that enables internalization of the viral capsid into the cytoplasm [10,11].

Licorice belongs to the family Leguminosae and is distributed all over the world. In China, it is mainly distributed in Xinjiang, Gansu, Shaanxi, and other northwest regions [12]. Licorice is one of the most widely used herbs in the world, and has a long history of application [13]. Polysaccharides are one of the main bioactive components of licorice. Studies have shown that Glycyrrhiza polysaccharide (GCP) has biological activities such as immune regulation [14] and antioxidant [15], antitumor [16], antiviral [17], and antibacterial properties [18]. Therefore, GCP has wide prospective applications. At present, research on GCP has mainly focused on its immunomodulatory activity; it has been reported that GCP can activate macrophages and dendritic cells [19,20]. GCP can also facilitate immune response by promoting the proliferation of lymphocytes [21]. However, the effectiveness of GCP in anti-PRV infection is still unknown.

In this study, we explored the antiviral effects of GCP on PRV infection and further explored its antiviral mechanisms. Our data showed that GCP exhibited better antiviral effects in the early stages of PRV infection, and its antiviral mechanism inhibited viral binding and internalization. Our findings suggest that GCP has potential for use as a treatment against PRV infection.

## 2. Materials and Methods

### 2.1. Cells and Virus

PK-15 cells were maintained in Dulbecco’s Modified Eagle Medium (DMEM; Sigma, Canada) containing 5% fetal bovine serum (FBS; Lonsa, Uruguay, South America). Vero cells were cultured in DMEM containing 6% FBS. All cells were cultured at 37 ℃ with 5% CO_2_. PRV XJ5, PRV NT, and PRV Ra were preserved in our laboratory.

### 2.2. Reagents and Antibodies

GCP ≥ 98% (Ultraviolet, UV) was purchased from Ci Yuan Biotechnology Co., Ltd. (Shaanxi, China), and was diluted to 50 mg/mL with phosphate buffer saline (PBS). We purchased 4′,6-diamidino-2-phenylindole (DAPI) (C1005), β-Actin mouse monoclonal antibodies (AF5001), and HRP-labeled goat anti-mouse IgG (H + L) (A0216) from Beyotime Biotechnology (Shanghai, China). Anti-gB mouse monoclonal antibodies and PRV-positive sera were prepared in our laboratory. FITC-conjugated goat anti-mouse IgG antibodies (AP124F) and FITC-conjugated rabbit anti-pig IgG antibodies (F1638) were purchased from Sigma-Aldrich (St. Louis, MO, USA).

### 2.3. Cell Viability Assay

The viability of PK-15 cells after GCP treatment was determined by using the Enhanced Cell Counting Kit-8 (CCK-8) (Beyotime, Shanghai, China), following the manufacturer’s instructions. In brief, PK-15 cells were cultured in a 96-well plate and incubated at different concentrations (100, 200, 400, and 600 μg/mL) of GCP at 37 °C for 24 h. Then the cells in each well were added to 10 μL of CCK-8 solution and incubated for another 2 h at 37 °C. Absorbance was measured at 450 nm and cell viability was expressed as a percentage of control cell viability.

### 2.4. Infectivity Assay

PK-15 cells were seeded onto 6-well plates and cultured at 37 °C. When the cell density reached 70–80%, the original culture medium was discarded, and the cells were washed thrice with PBS. Then the cells were pre-treated with GCP (100, 200, 400, and 600 μg/mL) in DMEM at 37 °C for 1 h, followed by infection by PRV XJ5 at a multiplicity of infection (MOI) of 0.1 at 37 °C for 1 h. After 1 h of infection, the infected cells were washed thrice with PBS and cultured in DMEM containing 2% FBS in the presence of corresponding concentrations of GCP at 37 °C for 24h. The antiviral activity of GCP was measured by cytopathic effects (CPE), a Western blot, 50% tissue culture infective dose (TCID_50_), and an indirect immunofluorescence assay (IFA).

### 2.5. Time-of-Addition Assays

Time-of-addition assays were performed. PK-15 cells were either pre-treated, co-treated, or post-treated with GCP (100, 200, 400, and 600 μg/mL) during PRV infection. In brief: (i) Pre-treatment: PK-15 cells were inoculated with GCP at 37 °C for 1 h and subsequently washed thrice with PBS to remove GCP, then the cells were infected with PRV XJ5 (MOI = 0.1) for 1 h and subsequently washed thrice with PBS to remove unbound viruses. (ii) Co-treatment: PK-15 cells were simultaneously treated with PRV XJ5 (MOI = 0.1) and GCP at 37 °C for 1 h, then the cells were washed thrice with PBS to remove GCP and unbound viruses. (iii) Post-treatment: PK-15 cells were infected with PRV XJ5 (MOI = 0.1) at 37 °C for 1 h and subsequently washed thrice with PBS, then the cells were treatment with GCP at 37 °C. All the above-treated cells were then further cultured at 37℃ for 24 h.

### 2.6. Viral Attachment, Internalization, Replication, and Release Assays

In the viral attachment assay, PK-15 cells were incubated with PRV XJ5 (MOI = 0.1) and GCP (100, 200, 400, 600 μg/mL) at 4 °C for 1 h, then the infected cells were washed thrice with ice-cold PBS and cultured in DMEM containing 2% FBS at 37 °C for 24 h.

In the viral internalization assay, PK-15 cells were infected with PRV XJ5 (MOI = 0.1) at 4 °C for 1 h and subsequently washed thrice with chilled PBS to remove unbound viruses, then the infected cells were inoculated with GCP (100, 200, 400, and 600 μg/mL) at 37 °C for 1 h to initiate internalization. After 1 h, the cells were washed thrice with citric acid (40 mM citric acid, 10 mM KCl, 135 mM NaCl, pH 3.0) followed by PBS to remove PRV bound to the cell surface. Then the cells were cultured in DMEM containing 2% FB at 37 °C for 24 h.

In the viral replication assay, PK-15 cells were infected with PRV XJ5 (MOI = 0.1) at 37 °C for 1 h, then washed thrice with PBS. Then the infected cells were incubated with GCP (100, 200, 400, and 600 μg/mL) at 37 °C. At 4 and 6 hpi, the cells were collected to quantify the viral DNA by quantitative real-time PCR (qRT-PCR).

In the viral release assay, PK-15 cells were infected with PRV XJ5 (MOI = 0.1) at 37 °C and at 24 hpi, then the infected cells were washed thrice with PBS and cultured with GCP (100, 200, 400, and 600 μg/mL) in DMEM at 37 °C for another 4 h. Then the supernatants were collected to quantify the viral DNA by qRT-PCR.

### 2.7. Western Blot

The collected cells were lysed by 2 × SDS-PAGE loading buffer and boiled in a metal bath (96 °C, 15 min). The samples separated by 12% SDS-PAGE were then transferred from the gel to the nitrocellulose (NC) membrane. The NC membrane was blocked in PBST with 5% nonfat milk for 2 h at room temperature. The NC membrane was washed thrice with PBST and then incubated with anti-PRV glycoprotein B (gB) monoclonal antibodies (1:200) or anti-actin monoclonal antibodies (1:5000) overnight, at 4 °C. The next day, the NC membrane was washed thrice with PBST and incubated with HRP-conjugated secondary antibodies (1:5000) for 2 h at room temperature. Finally, an enhanced chemiluminescence (ECL) reagent (Share Biotechnology, Shanghai, China) was used to analyze the banding results of the immune complexes.

### 2.8. Viral Titer Assay

The viral titers were evaluated by the TCID_50_ assay. Vero cells were cultured with DMEM containing 6% fetal bovine serum (FBS) and inoculated in 96-well plates. The collected virus samples were diluted from 10^−1^ to 10^−7^ in DMEM successively. Then the diluted virus samples were inoculated to cells at 37 °C for 1.5 h. Finally, the virus-DMEM mixture was removed, and the infected cells were cultured in DMEM containing 2% FBS at 37 °C. After 72 h of culturing, the CPE on Vero cells were counted by a microscope and the TCID_50_ was calculated by the Reed-Muench method.

### 2.9. IFA

Cells were immobilized with 4% paraformaldehyde at 37 °C for 30 min, then incubated with 0.1% Triton X-100 at 37 °C for 10 min, followed by blocking with 5% BSA overnight at 4 °C. The next day, cells were incubated with PRV-positive serum (1:200) at 37 °C for 1 h, followed by incubation with FITC-conjugated rabbit anti-pig IgG secondary antibodies at 37 °C for 30 min. Finally, cells were stained with DAPI for 7 min and the results were observed with a fluorescence microscope.

### 2.10. qRT-PCR

Total DNA from cells or cell supernatants were extracted using the phenol chloride method [22].Viral DNA was quantified by qRT-PCR. In brief, the standard curve of each experiment was constructed with PRV-gB standard plasmids. The primers were as follows: 1) gB-F: ACAAGTTCAAGGCCCACATCTAC and 2) gB-R: GTCCGTGAAGCGGTTCGTGAT. The conditions were as follows: 95 °C for 60 s, followed by 45 cycles of 95 °C for 10 s, then 62 °C for 20 s. The copy number of virus genome DNA was calculated according to the CT value of the sample and the standard curve.

### 2.11. Statistical Analysis

All results were obtained from three independent experiments and were presented in means ± SD. All experimental groups were compared with the 0-concentration group. All data were analyzed using GraphPad Prism 8.0 via one-way ANOVAs. Significance levels were as follows: ns, not significant; * *p* < 0.05; ** *p* < 0.01; and *** *p* < 0.001.

## 3. Results

### 3.1. Toxicity of GCP toward PK-15 Cells

**Figure 1:**
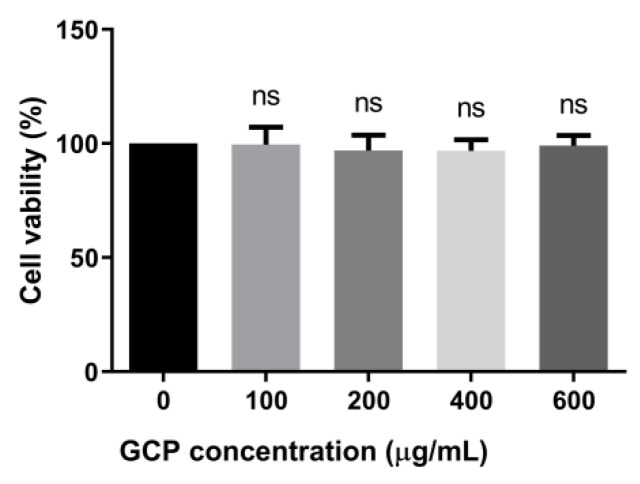
GCP cytotoxicity in PK-15 cells was detected with the Enhanced Cell Counting Kit-8. PK-15 cells were cultured in the presence of different concentrations of GCP for 24 h, and then added with 10 μL CCK-8 solution for another 2 h. Absorbance was measured at 450 nm (ns, not significant).

The cytotoxic effect of GCP on PK-15 cells was determined by the CCK-8 method after 24 h co-incubation; the concentrations of GCP were 100, 200, 400, and 600 μg/mL. Results revealed no cytotoxic effects of GCP from 100 to 600 μg/mL (compared with the 0 concentration group) (Figure 1). Therefore, GCP concentrations of 100 to 600 μg/mL were selected for the following experiments.

### 3.2. The Inhibitory Effect of GCP on PRV

**Figure 2:**
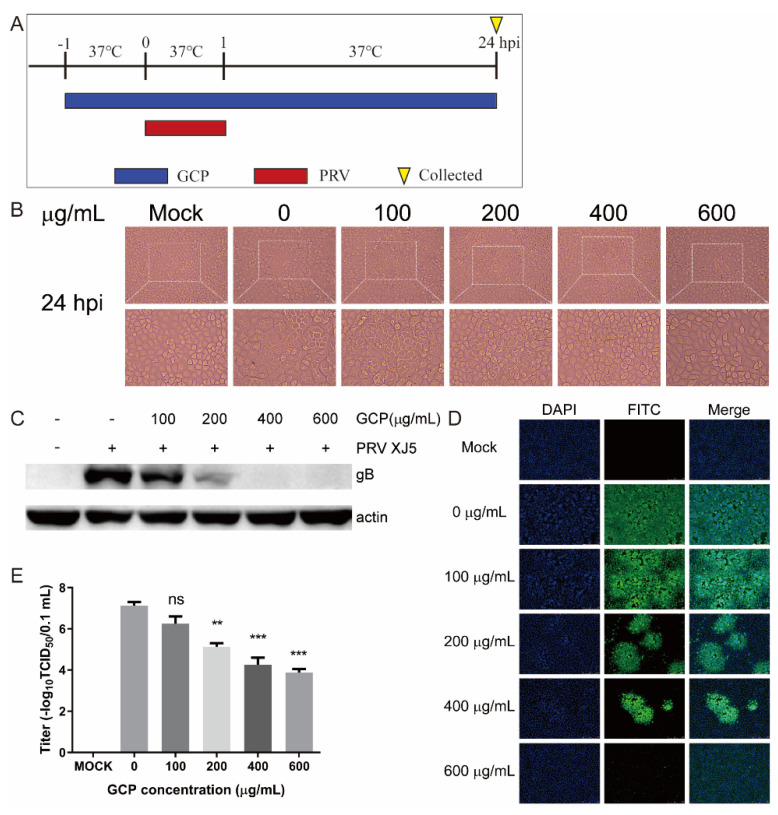
GCP inhibited PRV infection. (**A**) PK-15 cells were treated with GCP for 1 h and then infected with PRV XJ5 (MOI = 0.1) for 1 h. Cells were washed thrice with PBS and then incubated with GCP for another 24 h. (**B**) CPE observed by a microscope. (**C**) Expression levels of gB protein and actin analyzed by Western blot. (**D**) The internalized virus was detected by immunofluorescence assay. (**E**) The viral titer in supernatants was quantified by TCID_50_. All results were obtained from three independent experiments and were presented in means ± SD (ns, not significant; ** *p* < 0.01; *** *p* < 0.001).

To examine the antiviral effect of GCP against PRV—infected cells, PK-15 cells were pre—treated with GCP at 37 °C for 1 h and then infected with PRV XJ5 (MOI = 0.1) for 1 h. The infected cells were washed thrice with PBS and cultured with GCP in DMEM containing 2% FBS at 37 °C (Figure 2A). At 24 hpi, the CPE caused by PRV infection was observed through a microscope. Figure 2B shows that GCP significantly reduced PRV—induced CPE. Moreover, Western blot, TCID_50_, and IFA assays were performed to further confirm this inhibitory effect. As shown in Figure 2C, the expression levels of the PRV gB protein showed a dose-dependent decrease, and no viral protein expression was observed at concentrations of 400 and 600 μg/mL GCP. The results of the TCID_50_ assay shows that virus titers in cell supernatants significantly decreased by GCP—treatment compared with control cells, and the decrease rate was about 45.6% at 600 μg/mL of GCP (Figure 2E). Similarly, IFA results also confirmed the inhibitory effect, and the higher the concentration of GCP, the stronger the inhibitory effect (Figure 2D).

**Figure 3:**
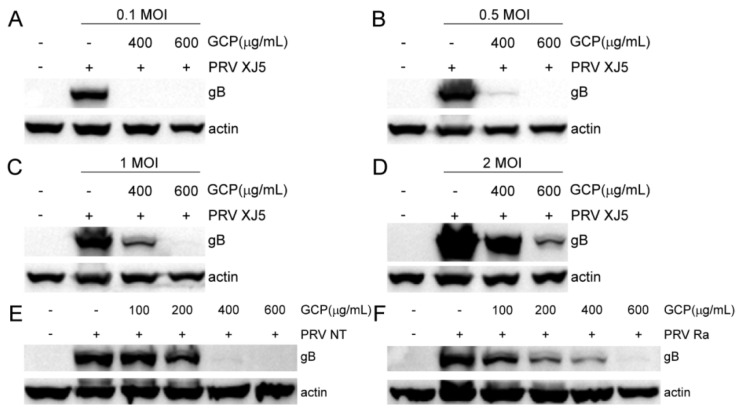
GCP reduced different MOI and PRV strains of infection. (**A**–**D**) GCP—preincubated PK-15 cells were incubated with PRV XJ5 at an MOI of 0.1, 0.5, 1, or 2. The expression levels of gB protein and actin were analyzed by Western blot. (**E**,**F**) GCP—preincubated PK-15 cells were incubated with PRV NT (MOI = 0.1) or PRV Ra (MOI = 0.1). The expression levels of gB protein and actin were analyzed by Western blot.

To rule out the possibility that the dose of virus infection had an effect, we performed assays with PRV XJ5 at an MOI of 0.1, 0.5, 1, or 2 in PK-15 cells as described above. Compared with the gB protein levels of the control cells, gB protein levels of the GCP—treated cells were significantly decreased at all tested MOI in PK-15 cells (Figure 3A–D), suggesting that the inhibitory effect of GCP on PRV infection was independent of the dose of virus infection. We further evaluated the antiviral effect of GCP on PRV NT and PRV Ra; we found that GCP had a strong inhibitory effect on different PRV strains (Figure 3E,F).

### 3.3. GCP Affects the Initial Stages of PRV Infection

**Figure 4:**
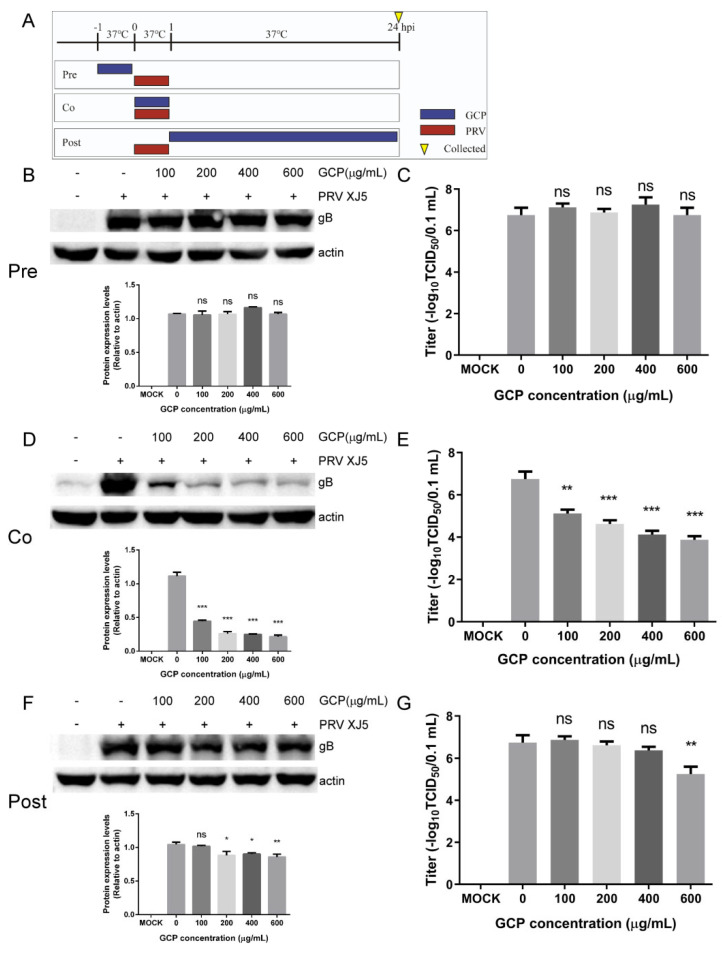
GCP inhibited the early stages of PRV infection. (**A**) Schematic diagram of GCP administration. PK-15 cells were infected with PRV XJ5 (MOI = 0.1) for 1 h and cells were treated with GCP at different hpi, designated pre—treatment (pre), co—treatment (co), or post—treatment (post). (**B**,**C**) Western blot for detection of gB expression and TCID_50_ for detection of viral titer after pre-treatment with GCP. (**D**,**E**) Western blot for detection of gB expression and TCID_50_ for detection of viral titer after co—treatment with GCP. (**F**,**G**) Western blot for detection of gB expression and TCID_50_ for detection of viral titer after post—treatment with GCP. All results were obtained from three independent experiments and were presented in means ± SD (ns, not significant; * *p* < 0.05; ** *p* < 0.01; *** *p* < 0.001).

We next investigated which stage of PRV infection was affected by GCP. Three different treatment schemes were used when adding GCP to the cells (Figure 4A). At 24 hpi, the virus titers in the supernatants of the infected cells were evaluated by TCID_50_. The expression levels of PRV gB protein were evaluated by Western blot. According to the results of the Western blot and TCID_50_, co—treatment demonstrated inhibition rates of about 80.6 and 42.6% at 600 μg/mL GCP, based on gB protein levels and virus titers (Figure 4D,E). Post—treatment showed inhibition rates of 17.7 and 25.9% at 600 μg/mL GCP, based on gB protein levels and virus titers (Figure 4F,G). Therefore, the inhibitory effect was most significant in GCP co—treatment with PRV. In contrast, pre—treatment of cells had no effect (Figure 4B,C). These results suggest that GCP plays an inhibitory role in the early stages of PRV infection.

### 3.4. GCP Inhibits PRV Binding

**Figure 5:**
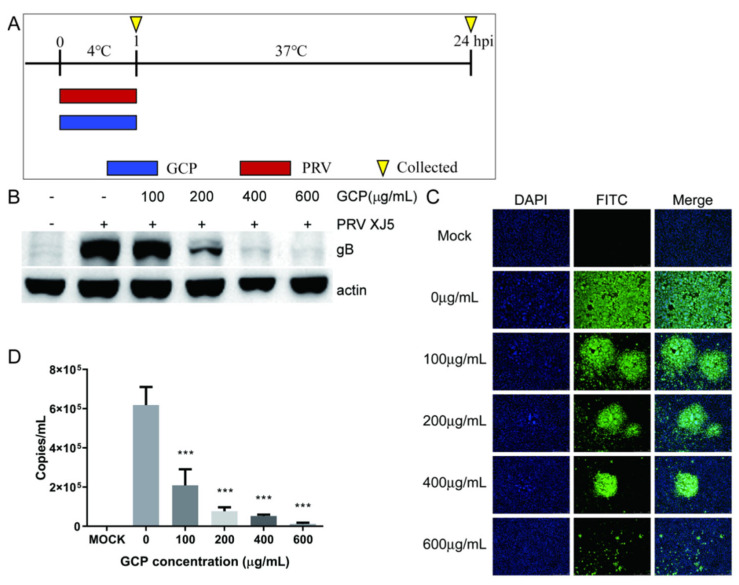
GCP decreased PRV binding (**A**) PK-15 cells were incubated with PRV XJ5 (MOI = 0.1) and GCP at 4 °C for 1 h, then cells were collected at 1 hpi or 24 hpi. (**B**,**C**) At 24 hpi, the expression levels of gB protein and actin were analyzed by Western blot and the internalized virus was detected by immunofluorescence assay. (**D**) At 1 hpi, the copy numbers of PRV DNA were quantified by qRT-PCR. All results were obtained from three independent experiments and were presented in means ± SD (*** *p* < 0.001).

Binding to the cell surface is the first step of early—stage PRV infection. So, we explored whether GCP inhibits PRV binding. PK-15 cells were incubated with PRV XJ5 (MOI = 0.1) and corresponding concentrations of GCP at 4 °C for 1 h, then the infected cells were washed thrice with chilled PBS and cultured at 37 °C (Figure 5A). At 24 hpi, PRV gB protein levels were determined by Western blot. We found that compared with control cells, the gB protein level of GCP—treated cells had decreased in a concentration—dependent manner, and 600 μg/mL of GCP almost completely blocked PRV binding (Figure B). In the same way, according to the IFA assay, GCP decreased the numbers of infected cells (Figure 5C).

To further verify these results, we performed a qRT-PCR assay to quantify PRV particles on the cell surface. PK-15 cells were processed as described above. At 1 hpi, the cells were washed with chilled PBS and collected to measure virus DNA copies by qRT-PCR. As expected, we found that the viral DNA copy level of GCP—treated cells had significantly decreased, which confirmed that GCP inhibits PRV binding to PK-15 cells (Figure D).

### 3.5. GCP Inhibits the Internalization of PRV

**Figure 6:**
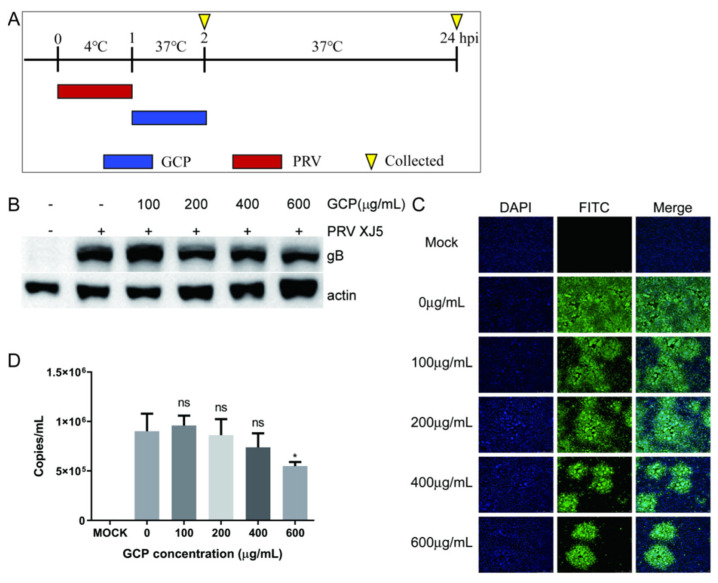
GCP decreased the internalization of PRV. (**A**) PK-15 cells were first infected with PRV XJ5 (MOI = 0.1) at 4 °C for 1 h, then incubated with GCP for another 1 h at 37 °C. (**B**,**C**) At 24 hpi, the expression levels of gB protein and actin were analyzed by Western blot and the internalized virus was detected by immunofluorescence assay. (**D**) At 2 hpi, the copy numbers of PRV DNA were quantified by qRT-PCR. All results were obtained from three independent experiments and were presented in means ± SD (ns, not significant; * *p* < 0.05).

PRV is internalized into cells through the viral protein-mediated fusion of the viral envelope and the cellular membrane. Therefore, we tested whether GCP inhibits the internalization of PRV following viral attachment in PK-15 cells. A schematic of this timeline is shown in Figure 6A. At 24 hpi, the cells were collected to determine the expression of the PRV gB protein. We found that expression of gB was reduced compared with the control group, with an inhibition rate of 43.2% at 600 μg/mL GCP (Figure 6B). Meanwhile, similar dose—dependent inhibition of virus internalization was observed by the IFA assay (Figure 6C).

We also performed a qRT-PCR assay to further verify these results. At 2 hpi, the washed cells were lysed for qRT-PCR to determine viral DNA copy levels. The results showed that GCP treatment inhibited PRV internalization into PK-15 cells; 600 μg/mL of GCP prevented 39.1% of virus internalization into cells (Figure 6D). Interestingly, the inhibition effect of GCP on PRV internalization was not as significant as that on PRV binding.

### 3.6. Effect of GCP on PRV Replication and Release

**Figure 7:**
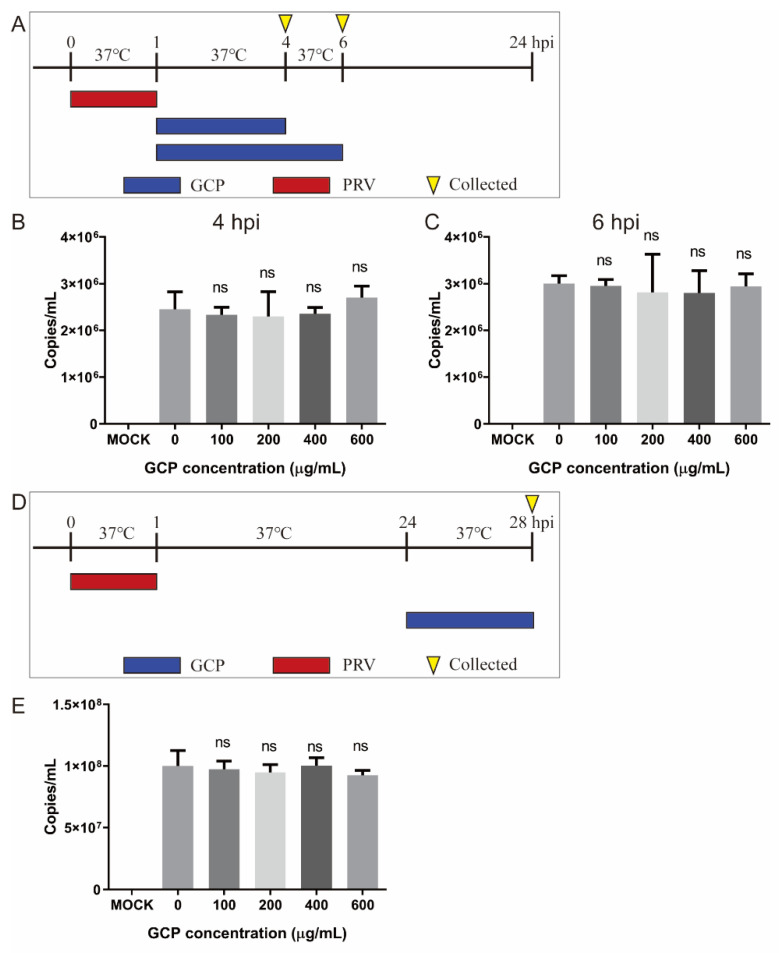
GCP had no effect on PRV replication and release. (**A**–**C**) PK-15 cells were infected with PRV XJ5 (MOI = 0.1) at 37 °C for 1 h, then incubated with GCP at 37 °C. At 4 and 6 hpi, the copy numbers of PRV DNA were quantified by qRT-PCR. (**D**,**E**) PK-15 cells were infected with PRV XJ5 (MOI = 0.1) for 24 h, washed, and incubated with fresh DMEM containing GCP for another 4 h, The copy numbers of PRV DNA in supernatants were quantified by qRT-PCR. All results were obtained from three independent experiments and were presented in means ± SD (ns, not significant).

For the viral replication assay, PK-15 cells were infected with PRV XJ5 (MOI = 0.1) at 37 °C for 1 h, then washed thrice with PBS. The infected cells were then incubated with corresponding concentrations of GCP at 37 °C. At 4 and 6 hpi, the cells were washed and collected to determine the viral DNA levels by qRT-PCR. We found that there was no significant difference in viral DNA levels between GCP—treated PK-15 cells and control cells, suggesting that GCP had no effect on PRV replication (Figure 7A,B).

For the viral release assay, PK-15 cells were infected with PRV XJ5 (MOI = 0.1) at 37 °C and at 24 hpi, then the infected cells were washed thrice with PBS and cultured with corresponding concentrations of GCP at 37 °C for another 4 h. The cell culture supernatant was then collected to measure the viral DNA levels. The results showed that GCP did not inhibit PRV release(Figure 7C,D).

## 4. Discussion

Vaccination is considered to be one of the most effective methods to control PR and reduce economic losses in the swine industry [5]. For nearly 40 years, the classic Bartha-K61 vaccine has been widely used to prevent and control the disease in China [23], but at the end of 2011, many vaccinated pig farms in China experienced outbreaks of PR [7,8]. Research suggests that this new outbreak was caused by a mutated strain of PRV [24,25]. More importantly, these PRV variants can infect humans and cause endophthalmitis and encephalitis [26,27]. In 2020, a PRV variant was isolated for the first time in a human case of acute encephalitis [28]. Therefore, it is necessary to develop an effective drug to control the transmission of these PRV variants. In this study, we verified the effective inhibitory effect of GCP on PRV infection in vitro for the first time.

PRV infection can be divided into four different stages: (1) Viral binding to the cell surface; (2) Viral internalization into the cell; (3) Viral genome replication; and (4) Viral release [10,29]. Each stage is a potential target for antiviral drugs. Studies have reported the effect of different stages of drug-resistance to PRV infection. Meclizine interfered with PRV entry and release [30]. Germacrone, (S)-10-hydroxycamptothecin, and resveratrol inhibited viral replication in the early stages of infection [31,32,33]. Quercetin interacted with the PRV gD protein to block virus adsorption and entry [29]. In this study, using the time-of-addition assay, we found that cells pre-treated with GCP did not cause a decrease in PRV infectivity, suggesting that cellular factors, including cellular receptors for PRV, may not be sensitive to GCP [34]. When GCP and PRV were added simultaneously, GCP showed strong antiviral activity, suggesting that GCP mainly inhibits the early stages of PRV infection.

The first step of viral infection is to bind to a receptor on the cell surface to cause stable and irreversible adsorption, resulting in the subsequent entry process [35]. Blocking virus adsorption to the cell surface can prevent viral infection more effectively than administering drug treatments after viral infection. At 4 °C, the virus can only be adsorbed on the cell surface, and cannot enter the cell. So, GCP was added at 4 °C to verify the effect of GCP on virus adsorption [36]. We found a significant decrease in virus adsorption on the surface of GCP-treated cells. When the virus was adsorbed to the cell surface, GCP could also inhibit virus internalization. However, the inhibitory effects on virus internalization were not as significant as those on virus adsorption. Sulfated polysaccharides, such as sulfated Chuanmingshen violaceum polysaccharides [37], can block the positive charges on the cell surface using the negative charge of its sulfate group, causing interference in the viral adsorption process [35]. It has also been reported that the anti-HIV activity of sulfated polysaccharides is induced by the electrostatic interaction between the negative charge of the sulfate group and the positive charge of HIV gp120 [38]. In addition, polysaccharides can compete with viruses to bind to cell-surface receptors, or bind to the viruses themselves, preventing the viruses from attaching to cells [39,40,41]. However, the mechanism of GCP inhibiting PRV adsorption and internalization needs further study.

To sum up, this study first reported the significant anti-PRV activity of GCP. GCP played an inhibitory role in the early stages of PRV infection and inhibited PRV infection by blocking viral adsorption and internalization. This study provides a potential therapeutic agent to be used against PRV infection. However, the antiviral effect of GCP in vivo remains to be further studied. In addition, GCP shows antiviral effects only when added simultaneously with the virus, which may limit its application.
